# The effects of probiotics on risk and time preferences

**DOI:** 10.1038/s41598-022-16251-x

**Published:** 2022-07-15

**Authors:** Aline M. Dantas, Alexander T. Sack, Elisabeth Bruggen, Peiran Jiao, Teresa Schuhmann

**Affiliations:** 1 Section Brain Stimulation and Cognition, Department of Cognitive Neuroscience, Faculty of Psychology and Neuroscience, Maastricht University, Maastricht, The Netherlands; 2 Maastricht Brain Imaging Centre, Maastricht University, Maastricht, The Netherlands; 3 Department of Marketing and Supply Chain Management, School of Business and Economics, Maastricht University, Maastricht, The Netherlands; 4 Department of Psychiatry and Neuropsychology, School for Mental Health and Neuroscience (MHeNs), Brain+ Nerve Centre, Maastricht University Medical Centre+ (MUMC+), Maastricht, The Netherlands; 5 Department of Finance, School of Business and Economics, Maastricht University, Maastricht, The Netherlands

**Keywords:** Neuroscience, Cognitive neuroscience, Decision

## Abstract

Animal models, human neuroimaging and lesion studies revealed that the gut microbiota can influence the interaction between the central and the enteric nervous systems via the gut–brain axis (GBA) and can affect brain regions linked to basic emotional and cognitive processes. The role of the gut microbiota in decision-making in healthy humans thus far remains largely unknown. Our study establishes a functional relationship between the gut microbiota and healthy humans’ decisions that involve risk and time. We conducted a between subjects’ placebo-controlled double-blinded design, with two groups and two sessions separated by 28 days, during which participants received daily doses of probiotics or a placebo. We investigated whether the prolonged and controlled intake of probiotics affects risk-taking behavior and intertemporal choices using incentivized economic tasks. We found a significant decrease in risk-taking behavior and an increase in future-oriented choices in the probiotics group as compared to the placebo group. These findings provide the first direct experimental evidence suggesting a potential functional role on the part of the microbiota-gut-brain axis in decision-making, creating a path for potential clinical applications and allowing for a better understanding of the underlying neural mechanisms of risk-taking behavior and intertemporal choices.

## Introduction

Our gut hosts a complex ecosystem of bacteria which plays a fundamental role on maintaining our health, nutrition, immune defenses and as more recently discovered, brain activity and behavior^1^. These gut microbiota form a complex system composed also by the central and enteric nervous systems, known as the microbiota-gut-brain-axis^2^. The proper ecological balance of the gut microbiota is known to affect brain development, cognitive performance^3^, mood, reactivity to stress and socialization^4–6^, and it even plays a role in certain psychopathologies^3,7–9^. Nevertheless, we still lack an understanding toward the effects of this system on decision-making. To address this, we conducted a double-blinded experiment in which we externally administered probiotics or placebo among healthy participants and established the causal relationship between probiotics intake and risky and intertemporal decision-making.

The study of the relationship between gut microbiota and decision making in animal studies has shown promising results. For instance, antibiotics-induced changes in the gut microbiota lead to increases in exploratory behavior^10^, while germ-free mice exhibit anxiety-like behavior and increased risk-taking behavior, which reverts to normal levels with bacterial colonization^11^. Furthermore, it was revealed that the *Bifidobacteria infantis*, commonly present in healthy intestines, plays an important role in tryptophan metabolism, influencing serotonin production^12^. In a similar vein, research with germ-free mice revealed increased concentrations of cortical dopamine and the influence of the gut microbiota on the myelination of frontal brain areas^13^. All these factors play important roles in high-order cognitive processes, which also includes decision-making^14–17^.

The results obtained in animal models have been successfully replicated in humans, demonstrating the relevance of the gut microbiota for brain development, important cognitive processes and behavior^5,18^. Studies with patients have demonstrated the clinical potential of GBA interventions^19^ but fewer studies have explored the role of the GBA in healthy participants’ cognition^3^.

Amongst the studies with healthy participants, relevant findings were obtained with the use of brain functional magnetic resonance imaging (fMRI). For example, it was identified that the *Bifidobacterium* concentration is positively correlated with the increasing connectivity of the frontal nodes of the default mode network, while the prevalence of *Prevotella_9* and *Bacteroides* is negatively correlated^20^. It was also shown that participants who received probiotics, compared to a control group, had decreased functional connectivity between the frontal pole and frontal medial-cortex during resting-state fMRI^12^, as well as a significant reduction in brain activity in sensory and affective regions, such as the insula, and increased activity in cortical regulatory regions, such as the DLPFC and MPFC, during a standardized emotional face recognition task^28^. Both the DLPFC and ventromedial prefrontal cortex (VMPFC) play a fundamental role in decision-making^21^ and are especially relevant to risk-taking behavior^22–26^ and intertemporal choices^21,27,28^, the focus of this study. Although these findings cited above point to a potential influence of the GBA in decision-making, this direct relationship is still unexplored. Therefore, we here investigate how changes in the gut microbiota can affect human decision-making in healthy participants and more specifically risk-taking behavior and intertemporal decision-making.

**Figure 1:**
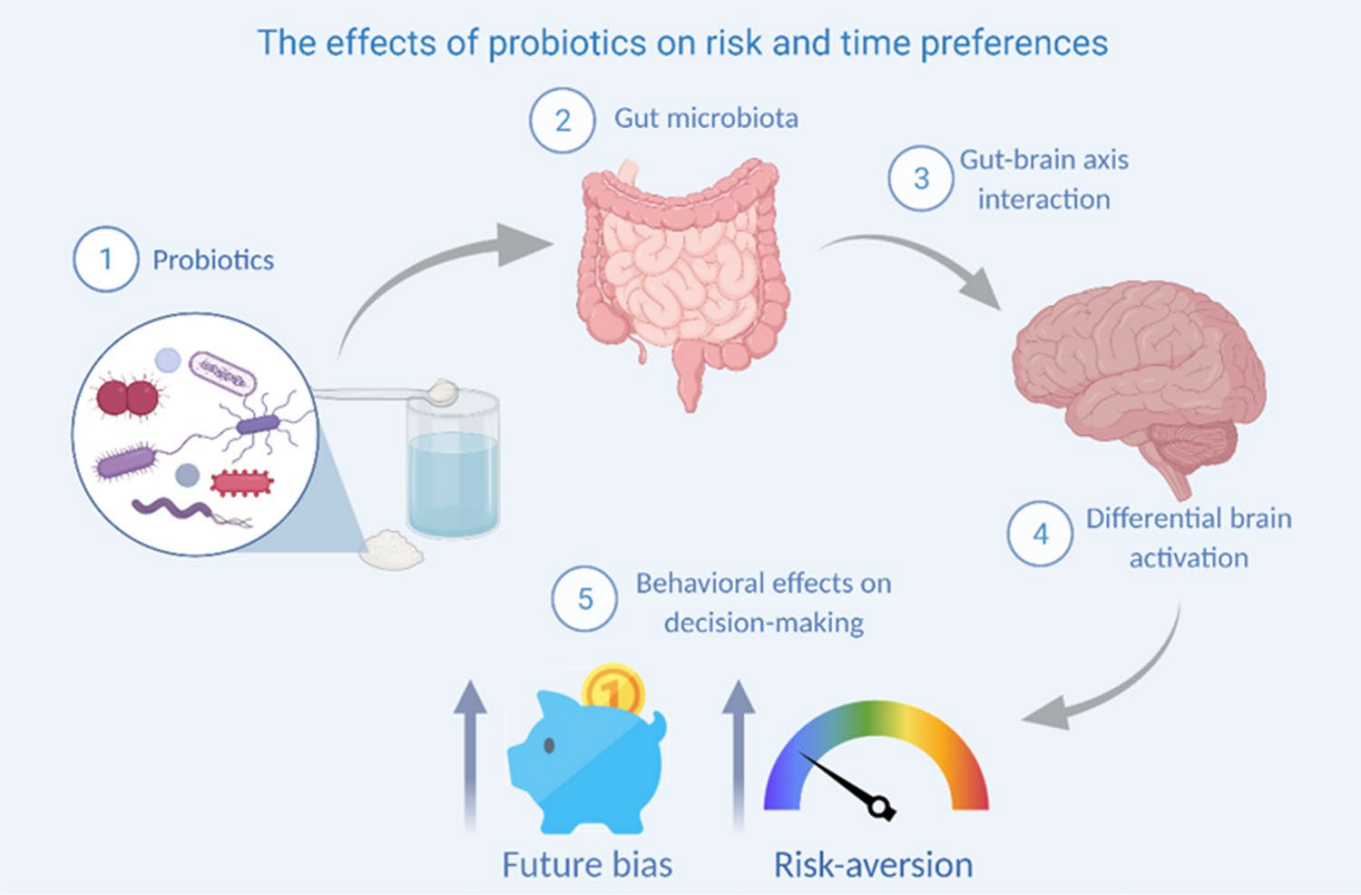
Graphic abstract. Figure depicts a graphic abstract of the present study.

To that end, we used a double-blind protocol with 4 weeks of ingestion of either probiotics or placebo (two groups, gender balanced, between subjects’ design) and incentivized tasks to measure risk-taking behavior and intertemporal choices. Specifically, we used the Maastricht Gambling Task (MGT)^24^ to measure risk-taking behavior and the Maastricht Choice Game (MCG) to assess intertemporal choices. We hypothesized that by changing the gut microbiota composition with the probiotics protocol, we could affect risk-taking behavior and intertemporal choices. These changes would occur via the gut-brain axis leading to changes in brain activity. According to the results observed both in animal models and the effects of probiotics in brain activity^8,11,18,29^, we expect a significant reduction both on risk-taking behavior and present bias. Hence, we hypothesized that the processing of risk and intertemporal decisions goes beyond the central nervous system’s limits in our healthy participants. A graphic abstract of our study is depicted in Fig. 1.

## Materials and methods

We conducted a double-blind between-subjects study, with probiotics or placebo administration as a between-subjects factor. The study included two experimental sessions with a 28-day interval, during which participants took daily doses of either active probiotics or placebo (see “Probiotics” for details). The study included a questionnaire to control for diet, arousal, self-control, and mood effects, as well as to provide alternative measures of time and risk preferences.

### Sample

We recruited 72 participants using posters on campus and social media targeting the local community. Due to the COVID-19 lockdown in March of 2019, twelve participants discontinued the experiment, and three participants were excluded from the sample because they did not follow the probiotic intake protocol. Therefore, 57 healthy adults, with no reported psychological, psychiatric or gastric disease, right-handed, using a gender balanced sample (29 women) and with an average age of 23.4 years (SD = 4) finished the experiment (29 in the probiotics group, 28 in the placebo group). All participants had normal or corrected-to-normal vision, reported to be well rested during the experimental session’s days and gave written informed consent after being introduced to the experiment and screened for safety.

The safety screening followed the procedures recommended by the manufacturer (Winclove probiotics, The Netherlands)^11,30^, excluding participants who had any sort of gastrointestinal disease or were using any medication during the experiment, with the exception of contraceptive pills. The study was approved by the local ethical committee, Maastricht University’s Ethics Review Committee of Psychology and Neuroscience (ERCPN, approval code OZL_208_15_05_2019) and carried out in accordance with the standards set by the Declaration of Helsinki (Fortaleza Amendments).

Participants were asked to not consume more than two units of alcohol a day or any drugs on the day before, as well as during the experiment. Additionally, they were required to not take any antibiotics, medication or other probiotics throughout the entire experimental period. Participants were compensated for participation and rewarded according to task outcome.

### Experimental design

**Figure 2:**
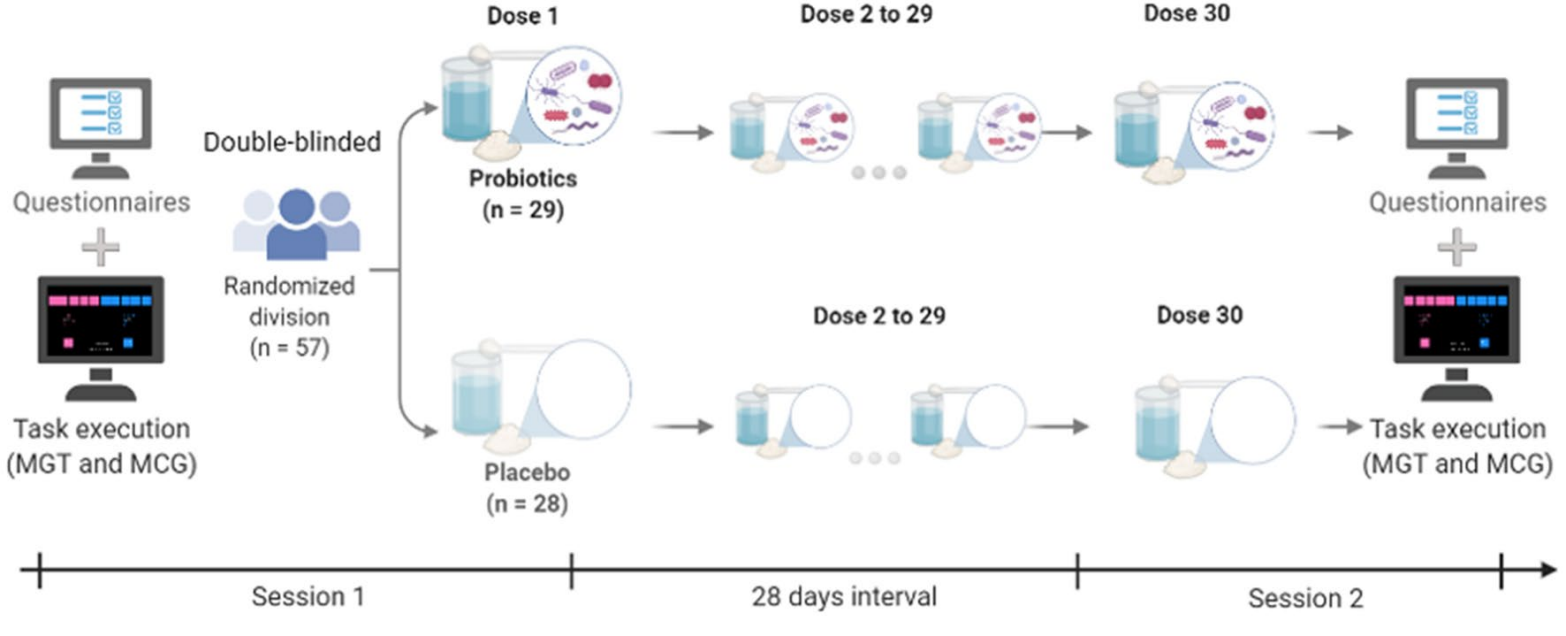
Experimental design. Figure depicts the double-blinded placebo-controlled experimental design, along with the main procedures for both experimental sessions and the probiotics/placebo protocol followed by the participants during the 30 days of the experiment.

Each participant underwent one of the assigned conditions of microbiota manipulation (probiotics or placebo). The conditions were assigned randomly, and the experiment was conducted in a double-blind fashion. Participation included two experimental sessions separated by 28 days (± 1), during which participants took daily doses of probiotics or placebo. Participants were reminded daily about probiotics ingestion via email to improve compliance, and a follow-up questionnaire was used in Session 2 to check for proper probiotic/placebo intake. The experimental design is illustrated in Fig. 2.

In each session, participants were invited to our laboratory, where they signed the written consent form and filled out a questionnaire via Qualtrics. They were instructed to not change their dietary patterns, not take any probiotics other than those provided to them as part of the experiment and not to take antibiotics during the 30 days of this experiment. Any deviations had to be reported, and participants who did not sufficiently comply with these requirements were excluded from the sample, with three participants being excluded from the sample for these reasons.

In the first session, participants first filled in a questionnaire to check for diet, arousal, self-control, and mood effects. We also used the Global Preference Survey^31^ (GPS) to estimate risk and time preferences. The survey was adapted using the text from the English version, with values pertinent to the Dutch population (based on the Dutch version), considering that our international participant base is fluent in English and resides in the Netherlands. After filling in the questionnaire, participants were also asked to fill out the Brief Self-Control Scale^32^ (BSCS), the Self-Assessment Manikin^33^ (SAM) and a short diet assessment. This was done to later be able to control for self-control, mood and potential dietary changes, respectively.

Finally, participants completed the Maastricht Gambling Task (MGT) and the Maastricht Choice Game (MCG), computer tasks used to elicit and estimate risk- -taking behavior and intertemporal choices, respectively. The task order was randomized to avoid any potential order effect. The explanation of each task was followed by ten practice trials before task execution. The tasks are described in more detail below.

After finishing the two tasks, we used an online random number generator, with which participants could select a random trial in each task that would be used to determine payments. Following the completion of the first session of the experiment, participants either received the first dose of probiotics or placebo and a box with the remaining 29 doses in individual sachets. They were instructed to take one dose daily for the next 28 days and reminded daily via mail to take their doses. The last dose was taken in Experimental Session 2.

To minimize possible differences in responses due to differences in the period of the day in which the experiment was conducted, session 2 took place on the 30th (± 1) day of the experimental period, at the same period of the day. Before starting the procedures, participants ingested the last dose of either probiotics or placebo at the lab. Participants also completed a check, in which they stated whether they missed any doses during the interval. Participants who missed taking three or more doses were excluded from the sample. The second session followed the same procedure as Session 1, only the GPS was now not administered. At the end of Session 2, participants were debriefed.

After each session, payments to participants were administered in two parts. In the first part, participants randomly chose one trial of the MGT and one trial of the MCG for payment. As the MCG involves payments at different dates after the session (explained in detail in “Maastricht Choice Game (MCG)”), bank transfers were made on the dates specified in a randomly chosen trial in MCG. We used this method so that all MCG payments could be done in the same way without participants having to come back to the lab. The second part of the payment, including their participation reward, at the rate of €7.5 per h, plus rewards to the randomly chosen trial in MGT was made using vouchers with monetary value. This was done at the end of the experiment (end of session 2).

### Maastricht Gambling Task (MGT)

**Figure 3:**
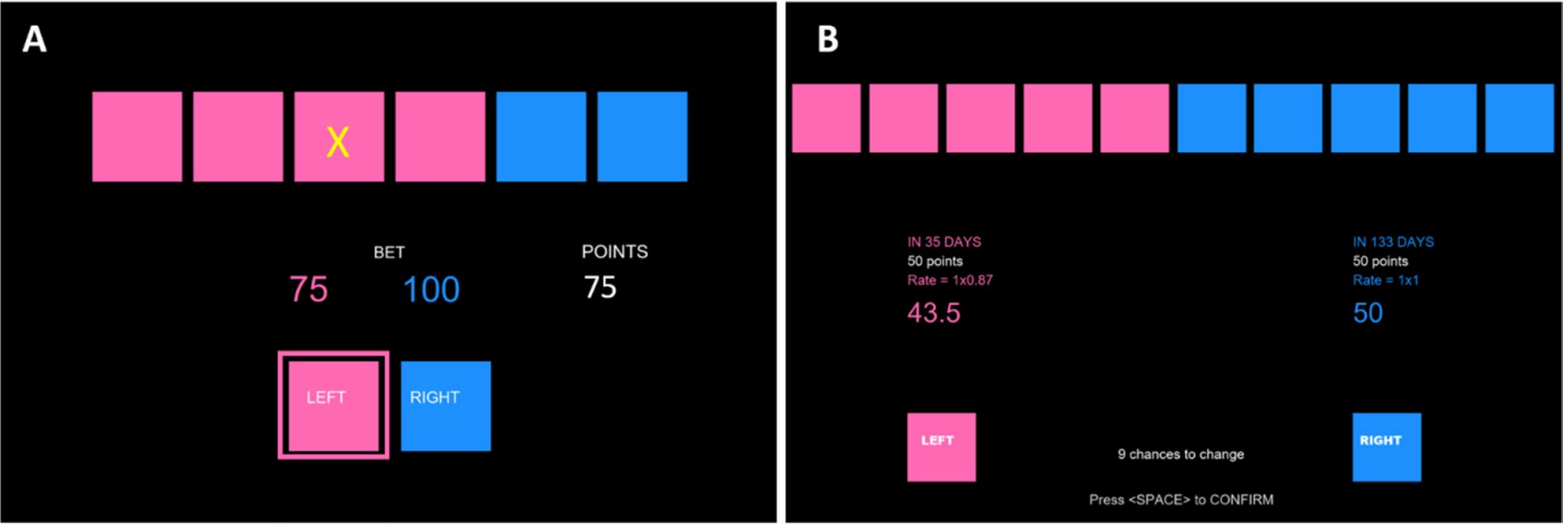
Example screens of the tasks used. (**A**) Displays an example screen from the Maastricht Gambling Task (MGT), and (**B**) an example screen from the Maastricht Choice Game (MCG).

The MGT is a computerized gambling task that is based on the Risk Task or Cambridge Gambling Task^34^ and further developed by Dantas et al. Participants are presented with six colored boxes (see Fig. 3A for an example screen) that can be either pink or blue. The number of pink boxes was randomized and could range from one to five (the remaining boxes are blue). Participants are informed that a token represented by a yellow X is hidden in one of the boxes. They need to guess the color of the box that hides the token. Unlike the original Risk Task, the MGT uses independent trials to control for memory effects. Moreover, to control for loss aversion, in the MGT participants do not lose points. The trials offer either positive points in case of a correct guess or zero points in the case of incorrect guess. Finally, to avoid any type of deception, all possible combinations of payoff and probabilities are presented to the participants and the token position is clearly randomized. Please see Dantas et al.^24^ for a complete description of the task.

### Maastricht Choice Game (MCG)

We developed the MCG to elicit and estimate intertemporal choices based on the Convex Time Budget (CTB) method developed by Andreoni and Sprenger^35^. We used the CTB concept applied in a similar choice environment that is used in the MGT to maintain a relatively constant visual stimulation. In contrast to the MGT, and following the CTB method, each participant is initially endowed with 100 tokens and must spend this endowment entirely on two options.

Each option involves a payment at a specific date, an earlier date and a later date. The earlier options (t) could either be immediate (0 days) or in 35 days (after the end of the experiment). The later options offered either 35, 72 or 90 days (k) after the earlier options (therefore, the later option date is t + k). Any amount allocated to the later option (t + k) stays the same, but those allocated to the earlier option (t) were multiplied by one of twenty potential discount factors (0.50, 0.525, 0.55, 0.575, 0.60, 0.625, 0.65, 0.675, 0.70, 0.725, 0.75, 0.775, 0.80, 0.825, 0.85, 0.875, 0.90, 0.925, 0.95 and 0.99). Therefore, 120 unique combinations of discount rates and dates were generated. Each combination was displayed twice, so a total of 240 trials were presented in a random order. These were divided into five blocks of 48 trials.

Participants could freely allocate the endowment between boxes of two colors. Pink boxes represented the earlier option, and blue boxes represented the later option. Each box represented 10% of the total endowment (10 points). Participants were limited to a maximum of 15 attempts before a final decision is made in each trial. The number of tokens allocated to each option and the payoff for each date were displayed on the screen. An example screen is presented in Fig. 3B.

Both MGT and MCG ruled out memory effects and wealth effects by using independent trials, in which the results of previous trials did not affect the following one.

To evaluate time preferences, we used the following model:

3 $$\underset{{x}_{t}}{Max} U\left({x}_{t}\right)= {{x}_{t}}^{\alpha }+{\beta }{\delta }^{k} {({x}_{t+k})}^{\alpha }$$

Such a model states that the choice in a trial is the result of utility maximization according to Eq. (3). In the model, *t* represents the earlier date (0 or 35 days), and *k* represents the delay between the earlier and later dates (35, 70 or 95). Therefore, ${x}_{t}$ is the payoff at date *t,* and ${x}_{t+k}$ is the payoff at date *t* + *k*. Parameter *α* captures risk attitude: $0<\alpha \le 1$; $\alpha =1$ indicates risk neutrality. Our estimation of *α* here provides an additional check for the results in our MGT regarding risk attitude. In order to deal with corner solutions, in which participants allocate all points to either the earlier or the latter option, our estimation strategy adopts the two-limit Tobit maximum likelihood regression^35^.

The parameters of major interest in this model for our research are each participant’s present-bias ($\beta >0$) and time discount ($0<\delta \le 1$). According to Andreoni and Sprenger^35^, $\beta <1$ indicates present-bias, while $\beta >1$ indicates future-bias. This parameter indicates how sharply a participant discounts between now and the immediate future. Finally, $\delta$ indicates a participant’s time discounting, or how much each dollar of future reward would be worth in present terms.

The compensation for this task was provided via bank transfer according to the trial randomly selected by the participant for payment. For the randomly selected trial for payment determination, bank transfer was done on the dates specified in that trial according to the allocation decision made in that trial.

### Probiotics

The probiotic Ecologic®Barrier (Ecologic®Barrier, Winclove probiotics, The Netherlands) is composed of Bifidobacterium bifidum W23, Bifidobacterium lactis W52, Lactobacillus acidophilus W37, Lactobacillus brevis W63, L. casei W56, Lactobacillus salivarius W24, and Lactococcus lactis (W19 and W58), distributed as sachets containing 2 g of freeze-dried powder of the PF and indicated for oral intake. Participants received the composition for 30 days^11,30^. For the same timeframe of 30 days, the control group received a bacteria-free placebo created by the same laboratory, which was based on corn starch and identical to the probiotic composition both visually and in flavor.

### Statistical analyses

To facilitate transparency and reproducibility, our datasets and codes are available at https://doi.org/10.17632/nbz385mhny.2. We analyzed the data from the MGT to estimate risk-taking behavior and the data from the MCG to estimate present-bias and time discounting.

All data were preprocessed using a custom MATLAB (The Mathworks Inc., Massachusetts, US). Our design included a between-subjects factor (group = placebo or probiotics) and a within-subject factor (time = session 1 and session 2). All trials (250 for the MGT and 240 for the MCG in each session) were analyzed per session and per participant. The control measures were analyzed with regressions using custom R scripts^36^. The intertemporal choice analyses included an extra step in preprocessing, in which the parameters $\alpha$ (risk attitude), $\beta$ (present-bias) and $\delta$ (time discounting) were estimated by running a two-limit Tobit maximum likelihood regression^35^. These parameters were estimated for each session.

The statistical analyses included a series of linear mixed model analyses, which are robust considering the missing data and appropriate for our mixed design. We again used custom R scripts^36^ to estimate the effects of the each factor and, more importantly, the interaction of time*group, which indicates the effects of the probiotics protocol versus the placebo protocol in Session 2. Our final models were fixed-effects models, with participant-specific and trial effects as the random effects. All the analyses presented normally distributed residuals and showed no heteroscedasticity.

Risk-taking behavior was analyzed by fitting a linear mixed model (formula = risk ~ group + time + group*time) estimated using REML. The follow-up analyses, including the payments received by the participants between sessions, were again estimated using REML, including the payments received as part of the MCG compensation (payment) and the participation fee from Session 1 (participation) (formula = risk ~ group + time + payment + participation + group*time).

The results of the MCG were again analyzed using linear mixed models. The effects of the probiotics protocol versus placebo on present-bias was estimated using REML, with group and session as the main factors. More importantly, we focus on the group*time interaction to evaluate the effects of the probiotics intervention (formula = present bias ~ group + time + group*time). The analyses of time discount were estimated with the same method and using REML (formula = time discount ~ group + time + group*time).

## Results

In this section, we present the main behavioral results of our experiment.

### Risk-taking behavior

**Figure 4:**
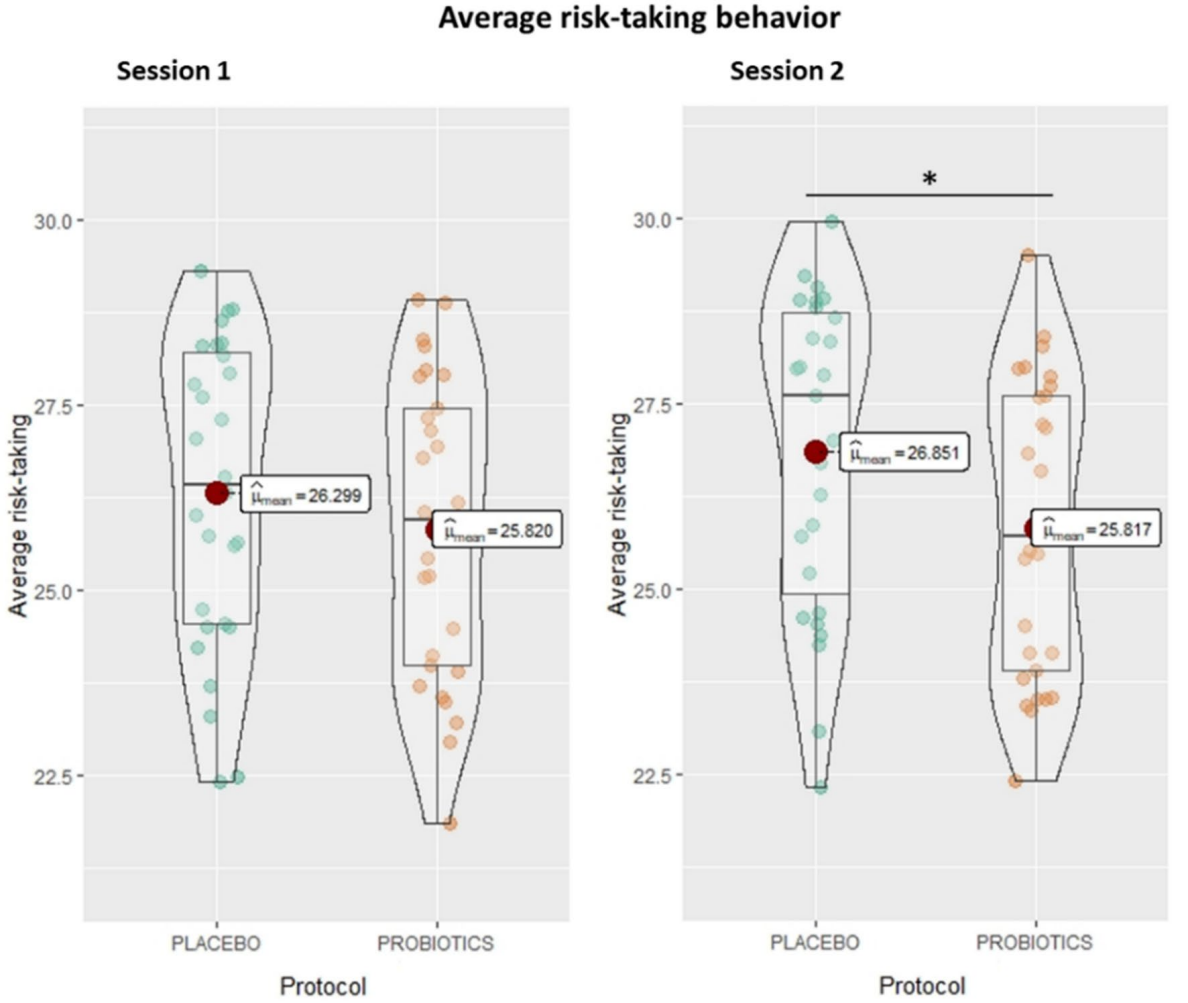
Average risk-taking behavior (n = 57). Average risk-taking estimated by the average standard deviation of each participant’s choice across sessions and protocols (placebo in green and probiotics in orange). The MGT allows risk scores from 1.8 to 50. The present analyses show participants’ average risk-taking scores. Average risk-taking scores vary between 21.84 and 29.31.

The interaction effect of group*time, which tests our hypothesis by comparing the effects on both groups after the probiotics/placebo intervention, is negative and can be considered small and significant (beta = − 0.42, SE = 0.16, t(14,118) = − 2.67, p = 0.008). This indicates that, despite the overall increase in risk-taking behavior over time, there was a significant reduction in risk-taking behavior in the probiotics group as compared to the placebo group in Session 2. More details can be observed in Fig. 4.

As expected, the effect of group was not significant (p = 0.922), indicating no difference between groups in the first session. There was a small positive and significant effect on the part of time (beta = 0.42, SE = 0.11, t(14,118) = 3.69, p < 0.001), indicating an increase in risk-taking behavior from Session 1 to Session 2 for both groups. To examine the observed increase in risk-taking behavior over time more closely, we ran an additional analysis. More specifically, we investigated whether the variation in the payout of the participant fee from the MCG task created a house money effect, or a payoff-based belief distortion^37,38^, and consequently increased risk-taking behavior^39^. We therefore added the payments received by participant between Sessions 1 and 2 to the model. These payments included the participation fee for all participants and the immediate payment of the MCG for some of the participants (others received it 35 days later, in line with the incentivized MCG task). The inclusion of two regressors for the amount of the immediate payment from the MCG (payment) and the participation fee (participation) significantly improved the model’s fit. The results yielded significant yet small effects on the part of the immediate payment (beta = − 0.04, SE = 0.02, p < 0.05) and the participation fee (beta = 0.03, SE = 0.12, p < 0.01). Our main result is robust to the addition of the two payment factors into the model, still indicating a significant negative effect on the part of the probiotics intervention, which can now be classified as a medium effect (beta = − 0.50, SE = 0.16, p < 0.01). Additional analyses for the MGT are available in the Supplemental material (S1).

### Intertemporal choices

**Figure 5:**
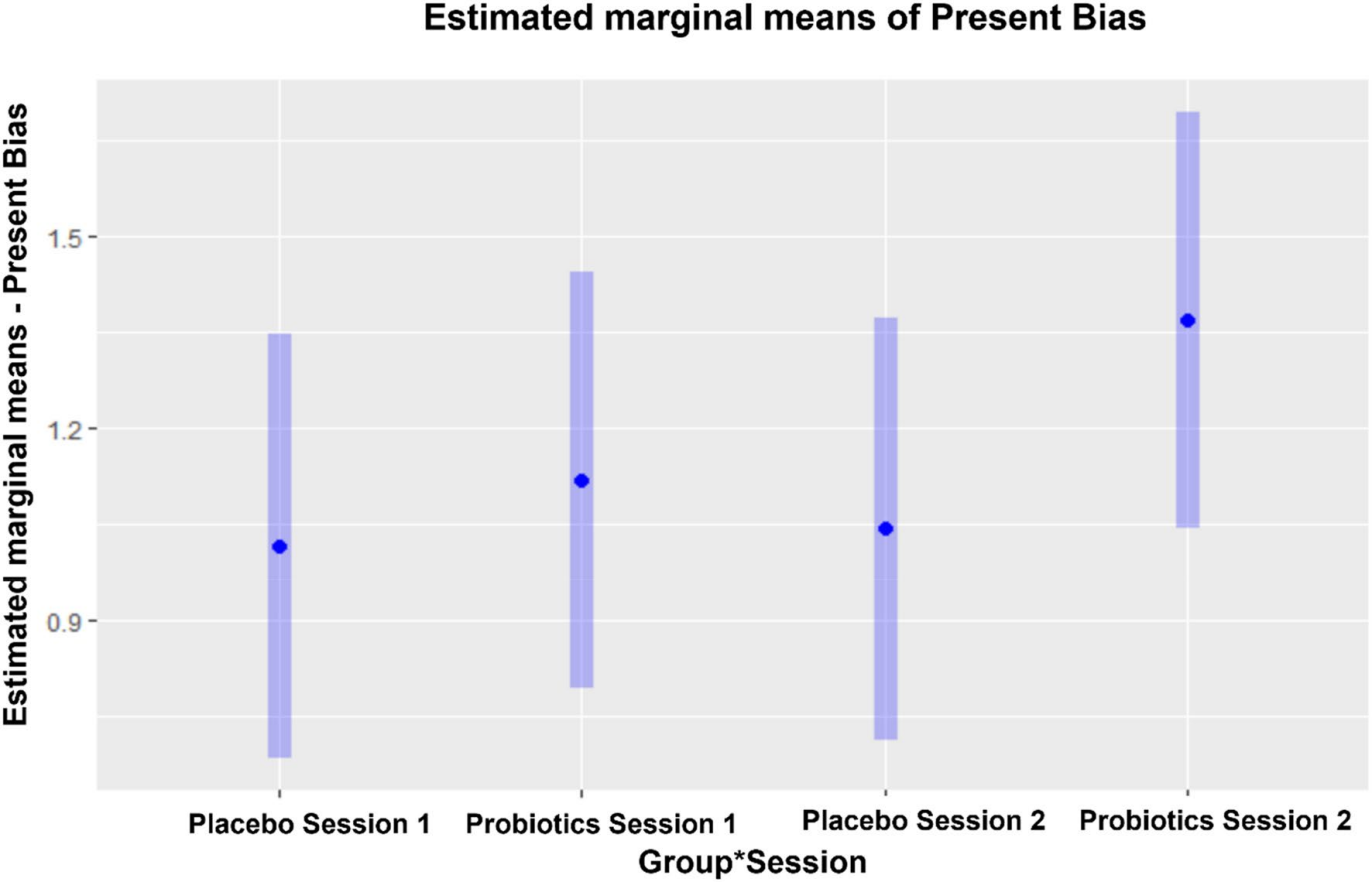
Estimated marginal means of Present Bias (n = 57). Estimated marginal means of present bias calculated using a linear mixed model considering as factors Time (Session 1 and Session 2) and Protocol (Probiotics and Placebo) and its interaction (Time*Protocol). Trial and Time are taken as repeated measures and participant-specific and trial effects are used as the random effects. Participants’ present bias (Beta) was estimated based on the model of convex budgets by Andreoni and Sprenger^35^ considering their responses during the MCG, with are averaged for each session. Dots represent participants’ estimated marginal means for each session and treatment. Bars indicate the 95% confidence interval of the linear model employed for data analyses. Values above 1 are interpreted as indicating future bias.

Regarding the probiotics interaction (group*time), we observed a significant large positive effect on $\beta$ (t(13,216) = 12.028, p < 0.001). This means that the probiotics intervention leads to a significant increase in $\beta$, to a value above 1, which, according to Andreoni and Sprenger^35^, is characterized as a future bias, meaning that these participants were more likely to make future-oriented choices. It is important to highlight that participants already presented $\beta$ values above 1, independent of the probiotics manipulation, indicating future bias, which is expected when using the convex time budget method^35^. Our results demonstrated a small, albeit significant, positive effect of session (t(13,261) = 1.99, p = 0.046), meaning that there was a small significant increase in $\beta$ from Session 1 to Session 2 in both groups. As expected, no significant effect of group was observed (p = 0.506). Details can be seen in Fig. 5.

Furthermore, we analyzed participants’ time discounting. The effect of the probiotics protocol, analyzed via the interaction group*session, was negative and can be considered medium and significant (beta = − 0.01, SE = 0.01, t(13,261) = − 4.911, p < 0.001). We did not find a significant main effect on the part of group, as expected (p = 0.24). There was a large and significant effect on the part of session (beta = 0.03, SE = 0.01, t(13,261) = 20.785, p < 0.001). Additional analyses for the MCG are available in the Supplemental material (S2).

### Control variables, time and risk preferences

We controlled for a series of variables, such as mood, self-control, arousal and diet. No significant effects on the part of the probiotics protocol (time*group) were observed on the mood scores (p = 0.17), self-control (p = 0.49), arousal (p = 0.72) or diet (p = 0.48). There were also no significant changes in diet when comparing the two time points estimated (p = 0.92).

We used the GPS^40^ to estimate participant’s time and risk preferences before the probiotics/placebo protocol, assuming a stability of this construct along time. These measurements were correlated to the task results aiming to find a correlation between risk preferences and risk-taking behavior (MGT results) and time preferences and intertemporal choices (MCG results).

Our results indicate a small significant correlation between risk-taking behavior estimated with the MGT and the GPS’s qualitative (r(14,123) = 0.03, p = 0.042) and quantitative estimation of risk preferences (r(14,123) = 0.02, p = 0.045). GPS’s qualitative measure of time preferences were negatively correlated with $\beta$ (present bias) (r(13,318) = − 0.04, p < 0.001) and no significant correlation with $\delta$ (time discounting). It’s quantitative estimation, named patience, was positively correlated with participants’ $\beta$ (r(13,318) = 0.04, p < 0.001) and $\delta$ a (r(13,078) = 0.33, p < 0.001). The visualization of such correlations are available in the Supplemental material (S1.4 and S2.3).

## Discussion

Given the crescent number of studies showing the fundamental relevance of the gut–brain axis as a bidirectional network in cognitive processes, here, we investigated the influence of the gut brain axis on decision-making in the face of risk and in the context of intertemporal choices^41^. To this end, we conducted a placebo-controlled double-blinded design with two sessions separated by 28 days, during which participants received daily doses of probiotics (or placebo). We investigated whether the prolonged and controlled intake of probiotics affected risk-taking behavior and intertemporal choices using incentivized tasks.

Our results confirmed the relationship between changes in the GBA and decision-making. Firstly, it was observed a significant reduction in risk-taking behavior after prolonged probiotic intake. Considering that there were no significant dietary or mood differences from Session 1 to Session 2 and the experimental conditions were identical, we can attribute the observed effects to the probiotic intake. Thus, participants who underwent the probiotics protocol were significantly less likely to choose risky options as compared to participants in the placebo group in Session 2, indicating a significant decrease in risk-taking behavior.

Secondly, our results showed that participants in the probiotics group exhibited a significantly higher future-bias and a significant reduction in time discounting as compared to the placebo group in Session 2. These results indicate that, after the prolonged use of probiotics, participants were significantly more likely to make future-oriented choices, investing more in delayed options than participants who received a placebo for the same period.

To further explore the robustness of our findings on risk-taking behavior, we control for additional factors. Since we observed that the placebo group exhibited a significant increase in risk taking in Session 2, we examined the results of the risk-taking behavior task more closely. We explored the potential reasons for this increase. We hypothesized that these increases in risk-taking behavior can be related to the fact that participants received the money between Session 1 to Session 2, potentially causing house money effect or payoff belief distortion^37,38^. The house money effect causes increases in risk-taking behavior in the presence of prior gains^37^, and payoff-based belief distortion, increases participants optimism and risk-proneness after a gain^37,38^.

This hypothesis was tested by adding the participation fee and immediate payments received between Session 1 and Session 2 as factors in our analysis. This way, we were able to show that the increase in risk-taking behavior in the placebo group was indeed an effect of the payments received by the participants between sessions; when we controlled for these payments in our model, the effect of time was no longer significant for either group. The effect of the interaction group*time, meaning the effects of the probiotics protocol on risk-taking after controlling for payments between sessions, not only remains significant but shows a larger effect size. Hence, we can affirm that the probiotics protocol led to a significant negative effect on risk-taking behavior. This means that, in the group that received probiotics, the significant increase in risk-taking behavior due to the payments between sessions seems to have been neutralized, considering that all other conditions were stable across groups and sessions.

In terms of intertemporal choices, we also observed an increase in future bias and time discounting from Session 1 to Session 2. These increases in both the placebo and probiotics groups are not unexpected and can be attributed to increased familiarity with the task and more confidence in the researchers, establishing a different reference point for their choices^11^. The probiotics intervention seems to have attenuated the effect on time discounting, which can be seen as a significant reduction in time discounting when comparing the probiotics and placebo groups in Session 2. Moreover, the group that underwent the probiotics protocol showed a larger significant increase in future bias than the placebo group, confirming the significant effect of probiotics on intertemporal choices.

Another interesting finding with respect to intertemporal choices is that participants were inherently future biased in both groups in Session 1, with an average β of 1.01, indicating future bias^35^. This contradicts the expectation based on the economics literature^47^, which holds that most people are present- rather than future-biased. However, deviations from present-bias are not uncommon in empirical studies^48^. Moreover, our results are in line with Andreoni and Sprenger^35^, who also use a convex time budget, as we do in our task. It is important to stress that our task already presents significant delay intervals and a wide variety of discount rates, which should lead to realistic representations of participants’ time preferences. One potential explanation for the future bias is that the payoffs offered to participant were not large enough, making it “easier” to wait for the payoffs^48,49^.

Overall, our findings about the effect on probiotics on risk-taking corroborate the results obtained in previous studies. According to research using animal models, germ-free rodents exhibit increased risk-taking behavior, which is reversed to a normal levels after their gut microbiota are normalized via fecal transplantation or probiotic administration^11,42^. The administration of the same probiotic composition (Ecologic®Barrier, Winclove probiotics, The Netherlands), for six weeks in rats led to a significant reduction in risk-taking behavior^11^. Regarding studies with humans, although it was not the main point of their study, Bagga et al. also observed a significant reduction in risk-aversion after four weeks of probiotics, in line with our findings^42^. Yet, this study used a different probiotics composition (Ecologic®825, Winclove probiotics, The Netherlands) and a non-incentivized, self-reported measure of risk.

To our knowledge, no study to date has explored how the GBA affects intertemporal choices. Roman et al. conducted a comparable study but used a two-choice task, which is considered a measurement of impulsivity rather than intertemporal preferences because the delay time is only 5 s^43^. Nevertheless, their results point in a similar direction as ours since the prolonged consumption of probiotics (3 weeks with daily ingestion of a milk yogurt containing *Lactobacillus casei Shirota*) led to a significant reduction in impulsive choices^43^.

Finally, it is important to highlight the potential practical impact of our current findings. Our results open doors for studies on the therapeutical use of probiotics in populations that present abnormal patterns of risk-taking behavior, such as patients with attention deficit and hyperactivity disorder (ADHD), addictions or depression^22,44,45^. Evidently, more studies in this direction are needed.

The communication between the gut and the brain during decision-making is also still unclear^3^. Two main pathways are potentially involved, namely the vagus nerve and neurotransmitter production^3,41^. Nevertheless, we can only speculate, at this point, that the changes in gut microbiota affect decision making through these pathways; the relative importance of each pathway in this neuronal network is still unclear^18^.

It is important to highlight that our study mainly focused on behavioral responses before and after the probiotics (or placebo protocol), in which participants were asked to confirm their compliance with the protocol verbally and completed a pre experimental check. Although these choices were based on published studies that used similar methods^4,5,8,42^, they also represent a limitation for our study, since no stool samples analyses were used to ensure differences in gut microbiota after the probiotics protocol and no additional measures were taken to evaluate possible metabolic changes due to the protocol. Therefore, we recommend the implementation of such steps in follow up studies.

In addition to potential clinical applications, the results that we observe for healthy participants calls for more research on the relationship between nutrition and decision-making. Various factors affect the gut microbiota, including genetics, health status, mode of birth, use of antibiotics, and stress levels^19^. However, diet is certainly one of the main factors to guarantee a balanced gut microbiota^46^. In our study, we used probiotics as a method to interfere with the microbiota-gut-brain axis by increasing microbiota diversity. Similar effects could potentially be achieved with a rich and healthy diet, healthier habits, and the lower use of unadvised antibiotics^7,47,48^. This is interesting in light of the fact that people with economic constraints often struggle to have access to nutritious diets^49^, which would facilitate higher risk taking and more present-bias. For example, participants with poorer diets could be more likely to prefer immediate consumption over investing in a pension plan, potentially compromising their future financial wellbeing, with significant financial, social and economic impacts.

In resume, our findings suggest that the gut-brain axis may be a fundamental player in the neuronal mechanisms underlying decision-making. This means that our current neuroeconomical models used to predict risk-taking behavior and intertemporal choices, among potentially other types of complex decision-making, should not be limited to the CNS.

## Supplementary Information

Supplementary Information.

## Data Availability

The data supporting the findings of this article, including scripts for statistical analyses are available at 10.17632/nbz385mhny.2.
