# Supplementary material for: The effects of probiotics on risk and time preferences
Source: Sci Rep. 2022 Jul 15;12:12152. doi: 10.1038/s41598-022-16251-x (PMC9287413; doi:10.1038/s41598-022-16251-x)

# Supplemental material

## Additional analyses

### S1. Maastricht Gambling Task – MGT (Risk-taking behavior)

#### S1.1 Average value

|  | **Value** | **Standard Error** | **DF** | **t-value** | **p-value** |
| --- | --- | --- | --- | --- | --- |
| (Intercept) | 59 | 0.92 | 14118 | 64.02 | <0.001 |
| Group | -0.08 | 1.29 | 14118 | -0.06 | 0.95 |
| SESSION | 0.98 | 0.26 | 14118 | 3.80 | <0.001 |
| Group*SESSION | -0.99 | 0.36 | 14118 | -2.77 | 0.006 |

#### S1.2Probability scores

|  | **Value** | **Standard Error** | **DF** | **t-value** | **p-value** |
| --- | --- | --- | --- | --- | --- |
| (Intercept) | 59 | 0.04 | 14118 | -20.09 | <0.001 |
| Group | -0.08 | 0.06 | 14118 | 1.72 | 0.086 |
| SESSION | 0.98 | 0.01 | 14118 | 1.63 | 0.103 |
| Group*SESSION | -0.99 | 0.02 | 14118 | -3.17 | 0.002 |

#### S1.3Response time

|  | **Value** | **Standard Error** | **DF** | **t-value** | **p-value** |
| --- | --- | --- | --- | --- | --- |
| (Intercept) | 1.29 | 0.04 | 13241 | 36.61 | <0.001 |
| Group | 0.12 | 0.05 | 13241 | 2.48 | 0.013 |
| SESSION | -0.17 | 8.36e-03 | 13241 | -20.90 | <0.001 |
| Group*SESSION | -0.07 | 0.01 | 13241 | -6.18 | <0.001 |

#### S1.4 Correlations with control scales

#### Correlations between the risk-taking behavior estimated using the MGT and estimates obtained from the scales used as control measurements. Pearson correlations (r) are displayed. *p<0.05; **p<0.01; ***p<0.001.


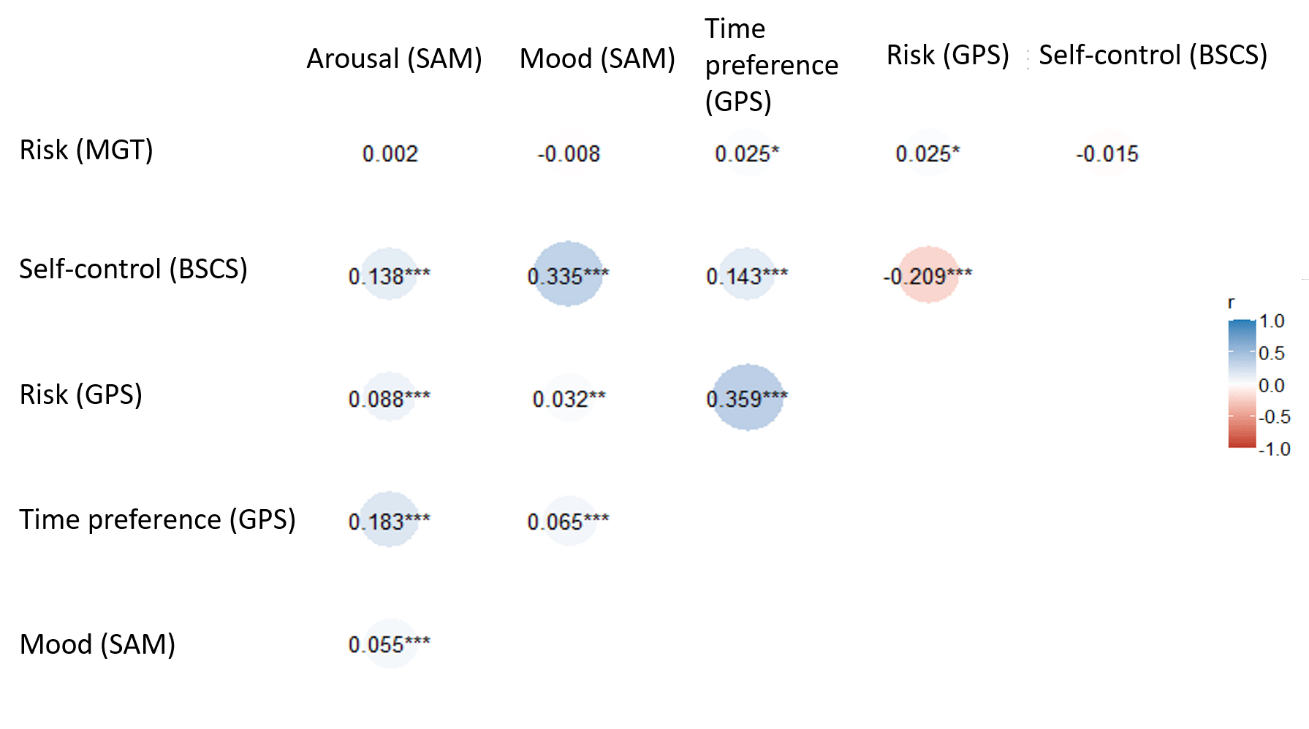


### S2. Maastricht Choice Game – MCG (Intertemporal choices)

#### S2.1 Risk attitude (α)

|  | **Value** | **Standard Error** | **DF** | **t-value** | **p-value** |
| --- | --- | --- | --- | --- | --- |
| (Intercept) | 0.90 | 0.21 | 13261 | 4.26 | <0.001 |
| Group | -0.42 | 0.29 | 55 | -1.42 | 0.16 |
| Session | -0.08 | 0.01 | 13261 | -9.89 | <0.001 |
| Group * Session | 0.13 | 0.01 | 13261 | 11.10 | <0.001 |

#### S2.2 Response time

|  | **Value** | **Standard Error** | **DF** | **t-value** | **p-value** |
| --- | --- | --- | --- | --- | --- |
| (Intercept) | -0.27 | 0.13 | 10979 | -2.16 | 0.03 |
| Group | 0.40 | 0.21 | 10979 | 1.87 | 0.06 |
| Session | -0.17 | 0.23 | 10979 | -0.69 | 0.49 |
| Group * Session | 0.22 | 0.17 | 10979 | 1.28 | 0.20 |

#### S2.3 Correlations with control scales

#### Correlations between the intertemporal choices estimated using the MCG and the estimates obtained with the scales used as control measurements. Pearson correlations (r) are displayed. *p<0.05; **p<0.01; ***p<0.001.


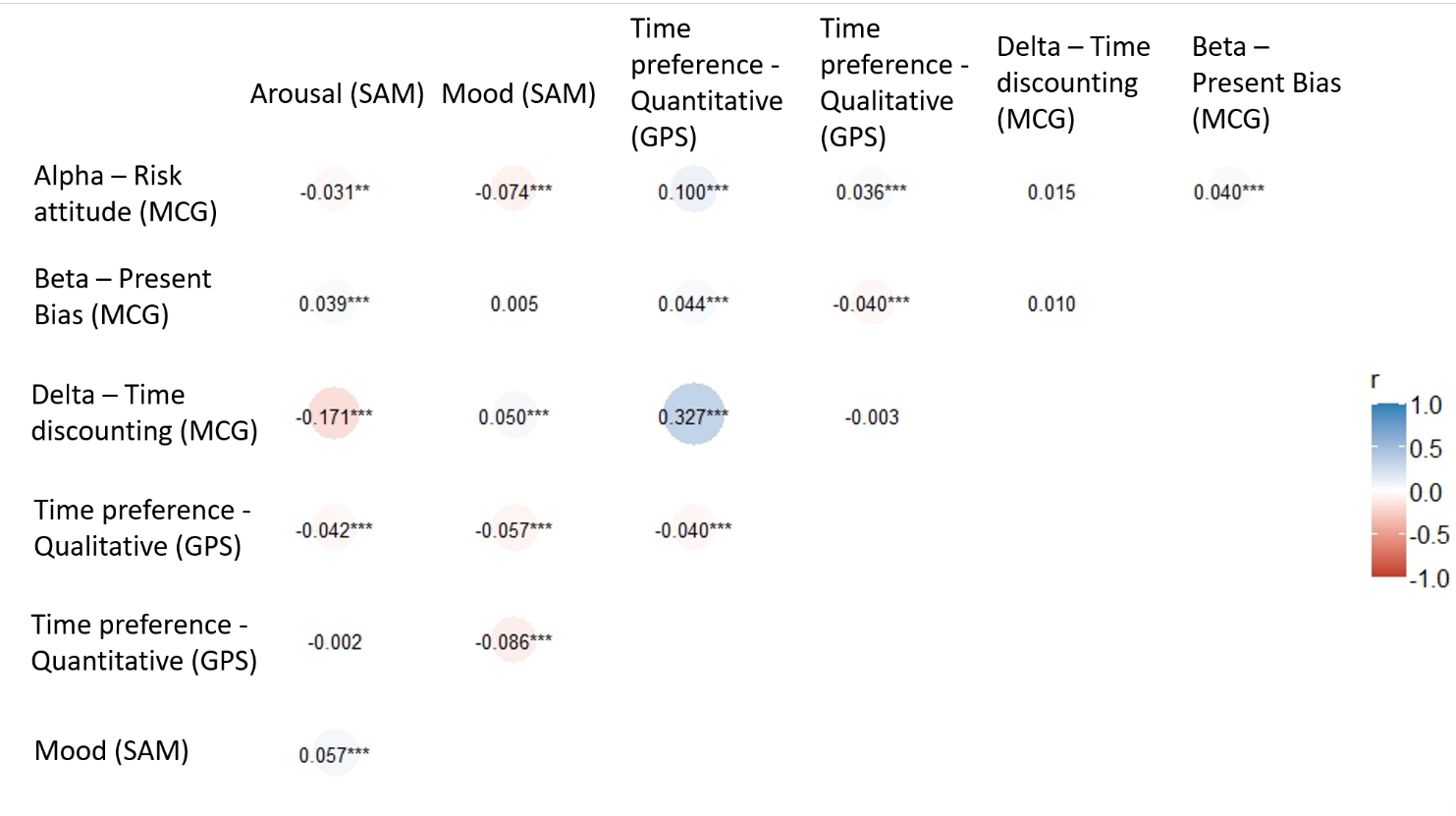

Supplement: Supplementary file 1 — Supplementary Information. [file 41598_2022_16251_MOESM1_ESM.docx]
